# Coproduction of a Theory-Based Digital Resource for Unpaid Carers (The Care Companion): Mixed-Methods Study

**DOI:** 10.2196/aging.9025

**Published:** 2018-02-28

**Authors:** Jeremy Dale, Joelle Loew, Veronica Nanton, Gillian Grason Smith

**Affiliations:** ^1^ Academic Primary Care Unit Division of Health Sciences Warwick Medical School Coventry United Kingdom; ^2^ Carers4Carers Warwickshire United Kingdom

**Keywords:** family caregivers, caregivers, self efficacy, information technology, frailty, Internet

## Abstract

**Background:**

Family and other unpaid carers are crucial to supporting the growing population of older people that are living outside residential care with frailty and comorbidities. The burden associated with caring affects carers’ well-being, thus limiting the sustainability of such care. There is a need for accessible, flexible, and responsive interventions that promote carers’ coping and resilience, and hence support maintenance of the health, well-being, and independence of the cared-for person.

**Objective:**

This study aimed to coproduce a digital program for carers to promote resilience and coping through supporting effective use of information and other Web-based resources. Its overlapping stages comprised the following: understanding the ways in which Web-based interventions may address challenges faced by carers, identifying target behaviors for the intervention, identifying intervention components, and developing the intervention prototype.

**Methods:**

The study was informed by person-based theories of coproduction and involved substantial patient and public involvement. It drew on the Behavior Change Wheel framework to support a systematic focus on behavioral issues relevant to caring. It comprised scoping literature reviews, interviews, and focus groups with carers and organizational stakeholders, and an agile, lean approach to information technology development. Qualitative data were analyzed using a thematic approach.

**Results:**

Four behavioral challenges were identified: burden of care, lack of knowledge, self-efficacy, and lack of time. Local health and social care services for carers were only being accessed by a minority of carers. Carers appreciated the potential value of Web-based resources but described difficulty identifying reliable information at times of need.

Key aspects of behavior change relevant to addressing these challenges were education (increasing knowledge and understanding), enablement (increasing means and reducing barriers for undertaking caring roles), and persuasion (changing beliefs and encouraging action toward active use of the intervention). In collaboration with carers, this was used to define requirements for the program.

A resources library was created to link to websites, Web-based guidance, videos, and other material that addressed condition-specific and generic information. Each resource was classified according to a taxonomy itemizing over 30 different subcategories of need under the headings Care Needs (of the cared-for person), General Information and Advice, and Sustaining the Carer. In addition, features such as a journal and mood monitor were incorporated to address other enablement challenges. The need for proactive, personalized prompts emerged; the program regularly prompts the carer to revisit and update their profile, which, together with their previous use of the intervention, drives notifications about resources and actions that may be of value.

**Conclusions:**

The person-based approach allowed an in-depth understanding of the biopsychosocial context of caring to inform the production of an engaging, relevant, applicable, and feasible Web-based intervention. User acceptance and feasibility testing is currently underway.

## Introduction

### The Importance of Caring

In the United Kingdom, there are approximately 6.8 million informal carers (ie, unpaid individuals who provide a combination of physical, practical, and emotional care and support), with an estimated value to the economy of over £130 billion per year [1,2]. With the increasing population of older people living with multiple morbidities, frailty, and other complex health and social care needs [1,3], the importance of the contribution of carers to society will continue to grow. In the United Kingdom, this has been recognized in policy documents and legislation such as the Care Act 2014, which recognize the need for information, guidance, and support to assist caring roles [4].

Carers face numerous challenges, from having to respond to the often complex physical, psychological, and social care needs of the cared-for person, living with the uncertainty and isolation that is frequently associated with caring, to finding relevant information and support [5]. Informal caring may be particularly challenging for older people who may themselves be frail and often are in a spousal relationship with the cared-for person, and for middle-aged women with multiple roles [3]. Although the majority of carers are of working age with a peak age of 50-64 years [6], the number over the age of 65 years is increasing rapidly. The amount of time spent caring increases with age, with the highest levels of commitment in people aged 80-89 years [6].

A recent UK survey reported that 21% of carers said that they received little or no helpful information or advice and felt they did not know where to go for support with caring, with a further 45% stating they received some but not all the information they needed [7]. Lack of support for carers is recognized as a contributory factor to unplanned hospital admissions, prolonged hospital stays, and delays in discharging patients [8-10]. There is also evidence that intensive caring (ie, for more than 20 hours per week) has a detrimental effect on the carer’s health [11]. Hence, there is a continuing and growing need for effective support for carers to undertake caring responsibilities [12], enhance their capacity to cope, and remain resilient [13].

Resilience is critical to sustainability as a carer and has been recognized as involving multidimensional individual factors, such as self-esteem, self-efficacy, skills and knowledge, family factors (such as supportive family relationships and resources), and societal elements (such as supportive social networks and access to resources), all of which may interact and change over time [14]. A recent realist review exploring strategies that enhance carers’ resilience and coping [15] proposed a framework of interdependent domains (extending social assets, strengthening psychological resources, ensuring timely availability of key external resources, maintaining physical health, and safeguarding quality of life) and found that interventions that most successfully enhanced resilience were those that utilized multi-domain components. For example, programs which combine tailored information with interaction among carers result in greater benefit to confidence, self-efficacy, stress, burden, and depression in contrast to those providing information alone [16,17].

### Digital Technology

Digital technology may have considerable relevance to addressing issues faced by informal carers, but there have been relatively few targeted interventions. In the United Kingdom, there has been considerable interest and investment in the use of assistive technologies [18] to support older people. This includes telehealth apps that support the management of long-term health conditions and telecare apps that may involve personal and environmental sensors in the home. However, the overall take up of such intervention remains relatively low, and little research has explored adoption and use of such technologies from the perspectives of informal carers. One recent study found that informal carers play a crucial role in supporting the patient’s decision to adopt and engage with such interventions, and concluded that efforts to increase adoption and engagement should adapt recruitment strategies and service pathways to support both the patient and their carer [19].

The internet and mobile apps are being increasingly used by carers to enable social interaction and provide access to information and advice that can support their caring role [16,20-22]. A benefit is ease of access from the convenience of the home without any need to leave the cared-for person [23-25], but barriers to uptake include lack of accessibility (eg, availability of a device that links to the internet), skills (eg, literacy and digital skills), motivation (eg, lack of awareness of the potential financial, social, and health benefits), and trust (eg, fear of crime, privacy, and knowledge of credible sources) [26,27]. Digital interventions that incorporate a personalized approach that is adaptive to ever-changing needs and issues are more likely to improve carers’ health outcomes [28,29]. Encouraging user-generated content helps to address the difficulty of ensuring that carers make continued use of the intervention [30].

Older age tends to be associated with more limited engagement with digital technology, and in terms of age group, the lowest rate of internet use is in those aged over 65 years, with nonusers reporting a lack of interest as the main reason for not using the internet [31]. However, take-up is rapidly increasing among older people [32], and in the United Kingdom, there are various schemes to encourage older people to go online and to help them encourage their digital capability. For example, third-sector organizations such as Age UK are developing older people’s understanding of the benefits of the internet and provide digital skills training and support through short courses for individuals or small groups, one-to-one tailored training, home visits, drop-in sessions, among others [33].

Although Web-based interventions and apps may significantly enhance carers’ access to information and advice, identifying Web-based resources that are up to date, reliable, easy to use, and relevant can be time-consuming and challenging, especially for those who have limited information technology (IT) literacy [33]. Hence, the aim of the study described here was to address this by developing an easy-to-navigate Web-based program for carers that would provide users with personalized information and resources relevant to their context and needs, and thereby promote coping and resilience of the carer and improved health, well-being, and independence of the cared-for person. The scope of the project was limited to considering the needs associated with caring for adults with frailty and long-term conditions that are associated with ageing, and to developing a full working prototype of the intervention.

## Methods

### Underlying Theory

This intervention development study draws on a theory-driven process of coproduction [34,35], and it involved substantial patient and public involvement together with input from policy makers, commissioners, health and social care providers, and voluntary sector organizations. We established a carers’ panel that included representatives of local carer support groups and Age UK, chaired by a carer and facilitated by the research team, and a stakeholder group with representatives of local health and social care commissioning and provider organizations, third-sector organizations, and voluntary organizations. Both groups contributed to all stages of the study. The study was also informed by the Behavior Change Wheel (BCW) framework [36,37], which was used to provide a systematic structured framework to focusing on behavioral issues relevant to caring that the intervention might address.

There were 4 key steps to the intervention development process: (1) understanding the ways in which Web-based interventions may address challenges faced by informal carers, (2) identifying and understanding target behaviors for the intervention, (3) identifying intervention components, and (4) developing the intervention prototype. Key aspects within each step are described below.

### Step 1: Understanding the Ways in Which Web-Based Interventions May Address Challenges Faced by Informal Carers

#### Scoping Review

To understand the challenges faced by informal carers and how these might be addressed through Web-based interventions, information was initially gathered through a scoping literature review that focused on review papers and policy documents. In addition to identifying the challenges faced by informal carers, key topics for the review included developments influencing the use of Web-based technology, guidance on the development of Web-based interventions, and consumer surveys on the use of the internet and mobile devices. Web-based databases and journals (eg, PubMed, *Chronic Illness*) were explored in addition to Google Scholar searches (see Multimedia Appendix 1 for search terms used).

#### Carer and Stakeholder Engagement

Carers and health and social care stakeholders were engaged in an iterative coproduction process over a 2-year period. This was conducted pragmatically to enable a broad range of individuals to contribute to the definition and refinement of ideas and themes.

Members of carer groups were invited to help us understand the experience of carers and their cared-for in relation to the challenges that are being faced and ways in which these might be addressed by a Web-based intervention. We used a topic guide that explored carers’ experience of using Web-based information and resources, their views about how a Web-based intervention might be of benefit in their caring role, and we discussed potential intervention components iteratively to establish their relevance and acceptability. Detailed notes were kept, and key themes were identified and tested at subsequent group meetings as part of the coproduction.

Groups were identified through a snowballing process that initially involved publicly available listings of local voluntary organizations, and through key stakeholders who used their contacts to offer opportunities to attend carer support groups, dementia cafes, and to arrange focus groups. As a result, 5 local carer support groups and 6 companionship groups with both the carer and cared-for in attendance (attendees ranging from 1-15) were involved in the coproduction, and we also held 1 informal focus group with 12 attendees recruited from carer support groups for a more in-depth discussion about the emerging themes. In total, over 60 individuals participated, all of whom were current carers, or had recent experience of caring for an older family member or friend.

Alongside the work with carers, over 20 managers and health care professionals from local authority stakeholders and National Health Service (NHS) Clinical Commissioning Groups (CCGs) were involved in mapping the findings from the literature review and the insights acquired from the engagement with the carer and cared-for community onto local and national policy and strategic planning. Particular attention was given to challenges around the limited information and support carers receive at crucial points along the caring pathway, and the potentially detrimental effect this has on quality of life and well-being. Specifically, we identified ways in which this intervention development study fitted with local projects, developments, and resources, and how it might enable improved health and the avoidance of NHS activity, including unplanned hospital and care home admissions.

#### Existing Platforms and Support

A range of platforms were identified that might be relevant to addressing the needs of the carer and the cared-for using search engines and reviewing websites currently offering support to carers and older people requiring care, provided by voluntary organizations, local authority, and NHS sources. A search of the NHS Apps Library, Apple iTunes store, and Google was performed to identify any relevant apps. Additionally, the UK Clinical Trials Database was reviewed to identify any relevant current trials involving relevant interventions. Platforms and websites were reviewed to identify gaps in scope and content and categorized into resources provided by charities or voluntary organizations, NHS or CCGs, local authorities, research projects, and private enterprises.

### Step 2: Identifying and Understanding Target Behaviors

Drawing on the literature review, focus groups, and stakeholder engagement, we identified key behaviors that might be targeted by the intervention based on feasibility and potential impact. The analysis of the behavioral challenges facing carers was reviewed at carer meetings, with both groups considering how they affected the carer’s resilience and their capability and capacity to cope. The BCW framework was used to help focus this process by identify aspects of the capabilities (C), opportunities (O), and motivation (M) that may enhance caring behaviors (B). This was done in conjunction with consideration of the Theoretical Domains Framework [36-38] and the domains known to be associated with carer resilience and coping [15]. These were refined as part of the carer engagement described earlier.

### Step 3: Identifying Intervention Components

Specific intervention functions (eg, education, prompting, and training) from the behavior change techniques proposed by the BCW were then identified together with candidate intervention components (ie, intervention functions, behavior change techniques) [36,37,39]. These were refined through ongoing discussion with carers and stakeholder organizations, as described earlier, to develop shared understanding of the priorities for the intervention development. A workshop was then held with user and IT design involvement to develop a list of user “wants” related to these challenges that might be addressed by the intervention.

### Step 4: Developing the Intervention Prototype

Operationalization of the selected intervention components into features emerged through a collaborative process involving an IT development company that utilized an agile, lean approach to programming, a panel of 5 carers who were recruited for their range of caring experience and then met monthly, and a study stakeholder group that met every 3 months. This process was informed by and facilitated by the research team. A user-story mapping approach was employed, whereby user stories informing intervention features were mapped out [40]. The emerging functionality was prioritized using the APEASE criteria (affordability, practicability, effectiveness and cost-effectiveness, acceptability, side-effects/safety, and equity) [36].

The panel of carers recruited from local support groups provided detailed input to the design of intervention features and content, reflecting their first-hand experience of carers’ needs, whereas the stakeholder group (representatives from local health service commissioning organizations, public health, social care, health providers, third-sector and voluntary organizations) ensured that provider and policy perspectives were also incorporated. The lean approach was intended to eliminate wasteful programming, and through applying user stories and multiple interim releases, the carers’ panel informed each stage of the prototype development.

### Data Analysis

We undertook a narrative synthesis of the key issues and concerns that were described, and then undertook a thematic analysis [41] of the focus group discussion and stakeholder engagement, synthesizing this with the literature review findings. We analyzed the data iteratively so that emergent findings could be tested and refined through further steps in the coproduction process and used to inform the prototype design. Initial broad themes, such as “carer experience” and “sources of support,” derived from the remit of the study were agreed by 2 members of the research team leading the analysis. Thereafter, the wider study team worked collaboratively to generate codes and to develop themes and subthemes through an iterative process. We validated the analysis through further discussion with the study’s carers’ panel.

## Results

Although the study design describes sequential steps, in practice, it was an iterative process that continued over a 2-year period. Here we summarize key elements that emerged from each step and together informed the design and development of the intervention prototype.

### The Potential for Web-Based Interventions to Address Challenges Faced by Informal Carers

The literature search produced 364 items, of which 74 were deemed to be relevant to the intervention development process. From synthesizing the key findings from the scoping review with the thematic analysis from the focus group discussions and stakeholder engagement, 4 behavioral challenges were identified: burden of care, lack of knowledge, self-efficacy, and lack of time.

#### Burden of Care

Carer burden describes the negative impact of caring on the carer’s physical, psychological, emotional, social, and financial situation [42-44]. It contributes to carers often neglecting their own health needs [45]. Our qualitative data confirmed that carers often feel isolated and alone and felt that there was an expectation that they would find relevant support (including access to resources and supportive social networks) themselves. Carer support groups offered by local voluntary organizations, while valued for their peer support and companionship by those who used them, were often viewed as lacking availability, accessibility, and relevance.

Local health service and social care strategies recognized the need for personalized support to minimize the burden of care and to enhance coping and resilience [46]. This included offering assessments to carers to determine whether they are eligible for financial support or carers’ breaks, as well as ensuring other services are in place, such as a telephone helpline, drop-ins, case workers, support groups, and training for carers provided by various third-sector organizations and smaller local voluntary organizations [46]. However, it was recognized that only a minority of carers accessed such services, and that barriers to access included limited hours of availability, location of services and activities, lack of transport, and difficulty in leaving the cared-for person [47].

#### Lack of Skills and Knowledge

Carers frequently describe having inadequate information regarding the health and social care needs of the care recipient and lack of knowledge of the support that is available and how to access it [48-51]. Focus group data confirmed that carers are often unsure about how to access support and useful information (from medical advice, financial and practical support to emotional support). Several participants stated that they tended to rely on informal advice and guidance from family and friends, or through talking to peers and sharing knowledge and experience.

Carers described a lack of support from health and social care professionals that was felt to continue across the entire caring pathway. This was identified as starting at the time of diagnosis, which was felt to be a crucial point where further support is needed, with insufficient consideration of likely care needs and how these might change over time. Although it was recognized that a great deal of information and advice is available, particularly Web-based, this was often perceived as being inaccessible or difficult to navigate, particularly under time constraints. Furthermore, the quality and reliability of information and advice was often unclear.

Three overarching categories of information and advice needs for carers were identified related to (1) the care needs of the cared-for person, (2) general information and advice, and (3) sustaining the carer. The literature review and focus groups were used to develop a taxonomy (Textbox 1) with over 30 items setting out key elements within each of these categories.

#### Lack of Time

Caring is time-consuming and can occupy the whole day and week. Many carers report that they are unable to take a break from their caring role [22]. Lacking time for themselves can exacerbate feelings of low mood, anger, and frustration [43,52,53]. It also contributes to social isolation, loss of relationships, and a narrowing of interests and activities. This was an important focus group theme, with many participants describing the difficulties of finding time both for themselves and for accessing resources and information that is useful to their caring role. The importance and value of taking time out and the need for respite was widely recognized, despite this being difficult to achieve.

#### Self-Efficacy

Self-efficacy is associated with mental and physical carer health, ability to cope with challenging situations, and the overall quality of caring provided [54-58]. Many focus group participants described having experienced a diminished sense of control over their lives when faced with the burden associated with caring and the challenges involved in determining and accessing support. In part, this was recognized as reflecting their own lack of knowledge and skills to identify and address diverse care needs. In addition, there were instances where carers described difficulty effectively communicating with health and social care professionals, which could exacerbate low self-efficacy. Only a minority of carers feel confident in accessing community services to help them provide care, handle condition-specific behavioral problems, or manage the frustrations of caregiving [59].

### Identifying and Understanding Potential Target Behaviors and Intervention Components

The key aspects of behavior change that emerged as relevant to the design of the intervention were education (increasing knowledge and understanding), enablement (increasing means and reducing barriers for undertaking caring roles), and persuasion (changing beliefs and encouraging action toward active use of the intervention). As shown in Textbox 2, many of the user “wants” that were identified at the workshop held with user and IT design input involved aspects of enablement and persuasion.

### Refinement of Candidate Intervention Features

Keywords, values, and concepts encompassing the candidate intervention components were weighted according to the scale of IT programming involved in developing them. All the elements were seen as important, and most emerged as priorities for a full working product, reflecting the diverse and varied challenges associated with caring and the difficulty of prioritizing one need over another. The IT designers, therefore, decided on the order of developing the functional elements in relation to cost and resource availability.

The name of the intervention was regarded as crucial in terms of representing its ethos and in engaging the population it was intended to serve. A short list of names was created with carers, and this was then tested in a survey with local Age UK members; 35 responses were received, and the name Care Companion emerged as the clear preference. This was felt to reflect the concept of a personalized resource that would act as a reliable friend providing elements of support, advice, and guidance, both proactively and at times of more urgent need, along the pathway of caring.

### Production of Intervention Prototype

Working with a Web design team, a minimum viable product that addressed the requirements identified in previous steps of the process was developed. Key requirements that emerged were that it should be intuitive to use, and hence accessible to people with limited IT literacy. It was designed to provide personalized access to information, resources, and advice according to the needs identified through the profile of the carer and the cared-for person. In addition to condition-specific resources aimed at increasing understanding about how to address varied and changing care needs, it would also contain more generic, locally relevant information and advice to help the carer navigate and gain access to the welfare and care system.

**Taxonomy developed for managing information needs.**

1. Care needs
  - Dealing with a diagnosis
  - Signs and symptoms
  - Therapies and treatments
  - Personal care
    - Eating and Drinking
    - Toileting
    - Washing
    - Moving and Handling
    - Physical activity
    - Exercising the mind
  - Practical aids and Adaptations
  - Transport
  - Housing
  - Relocating to a care home
  - Returning home from hospital
  - End of life care
    - After death
2. General information and advice
  - Confidentiality
  - Communicating with health and social care
  - Financial help and benefits
  - Legal affairs
  - Services and support
  - Planning ahead
  - Safeguarding
  - Work
  - Education and training
3. Sustaining the carer
  - Emotional support
  - Local support groups
  - Respite
  - Taking a break
  - My physical health
  - My mental health
  - Relationships
  - Living with loss and bereavement

**Carer “wants” identified for the intervention, with the key intervention functions shown in parentheses; asterisked elements were identified as higher priority.**

As a carer I want….

- trustworthy, locally relevant information, advice, and support to be available 24/7 (Education, Enablement)*
- to be prompted to do useful things *(Education, Enablement, Persuasion)*
- the statutory assessment to be at the forefront of my initial to-do-list so that I find out what entitlements, and so on, we are eligible for (Education, Persuasion)*
- to be carefully prompted to “Look to the Future” (eg, lasting power of attorney) so that I feel better prepared (Enablement, Persuasion)*
- key information tips and things to look for (eg, warning signs) based on the profile of the person that I care for (Education, Enablement)*
- tips and advice relevant to my situation so that I am better able to tailor my care (Education, Enablement)*
- a prepopulated list of relevant contacts that I can add to so I can quickly get hold of people when necessary (Enablement)*
- to have a “saved” section for information I find useful so that I can easily use it again (Enablement)*
- to browse Web-based content by condition/location/type/tags and highly rated so I can find relevant information easily (Enablement)*
- to bookmark resources so I can access them later (Enablement)*
- to learn from other carers’ experiences about how they have coped with similar challenges to those that I am facing *(Education, Enablement)*
- to be asked about my well-being so that the system can monitor my well-being (Persuasion)*
- to be asked about the well-being of the person that I care for so that the system can monitor their well-being and proactively provide me with advice or guidance (Education, Enablement)*
- to be prompted to read information or seek help if my mood changes or is persistently negative so that I am cared for too (*Persuasion)* *
- to see automatically generated notifications about new resources and information relevant to me so that I’m kept abreast of additions to the site
- to be able to record key events or incidents in my journal with a date and time (Enablement)*
- to be able to view previous entries chronologically so that I can see trends *(Enablement)*
- to print entries from my journal between specific dates in a clear and legible manner so I can use them to help me communicate with health and social care professionals *(Enablement)*
- to have an area on the site aimed at giving me a break so that I am able to have moments for myself (Persuasion)*
- a frequently asked questions (FAQs) section so I can better understand how to make the most of the site (Education, Enablement)*
- a glossary so that I can understand the language used in the context of caring *(Education)*

**Criteria used for assessing the quality of materials being considered for inclusion in the resources library. Quality assessed with the following scale: 1 (very poor); 2 (poor); 3 (acceptable); 4 (good); and 5 (very good).**

- Presentation: Is information presented in a clear and concise format? Is the source of information free from adverts and pop-ups?
- Coverage: How well does the information cover the topic of the resource? Is it clear who the information is aimed at?
- Accuracy: Is the information consistent with that you believe to be true? Is the information verified by other sources?
- Currency: Is the information up to date? When was it initially uploaded and has it been recently reviewed?
- Accessibility: Is the information freely accessible? Is the source of information easy to access and navigate through?
- Readability: How easy is it to understand what is written? Is the information communicated effectively? Does the source avoid excessive jargon and technical terms?
- Objectivity: How objective is the source? Does the information attempt to coerce or influence the reader?
- Authority: Is the source of the information a genuine authority on the subject? Is the information mostly fact rather than opinion?

Alongside the IT development work, a group of medical students, all of whom had personal experiences related to caring, collaborated with the carers’ panel on establishing a systematic approach to identifying webpages and resources to be linked to the intervention’s resources library. This included drafting brief descriptors of each resource (1 or 2 sentence lay language summaries). The resources were tagged according to the conditions that they related to and the taxonomy described in Textbox 1. A quality assurance process was developed drawing on established systems [60-62] which, together with peer review, ensured that there was a transparent auditable trail to account for the content included in the intervention’s library (see Textbox 3). Only resources that scored at ≥4 on all measures were considered for inclusion, and where several alternative resources were available, the highest scoring items were selected. The carers’ panel emphasized this as being important, given the difficulty of knowing the trustworthiness of resources identified by standard search engines. Initially, we focused on incorporating resources relevant to caring for a person with dementia, given the prevalence, importance, and complexity of this condition, and then broadened the content to cover other long-term conditions associated with frailty.

In this way, the resource library was designed to provide signposting to information, guidance, video, and other resources according to transparent quality standards, with an underlying system of classification that would support powerful search, view, and retrieval capabilities. Sections aimed at improving well-being and coping, such as “take a break” tips, ideas, and resources, were specifically aimed at reducing the burden of caring and maintaining carer resilience.

In addition to the resources library, the intervention includes a journal in which the carer can record day-to-day key information about the cared-for person (eg, regarding contacts with health care professionals, changes in condition) and a mood monitor. Changes that occur over time in self-ratings recorded in the mood monitor may drive messages to the user, such as encouragement to seek professional advice. There is also a “useful contacts” directory that can help the carer access support when needed, such as from local authority and third-sector organizations.

A further aspect is that the Care Companion should be dynamic and learn from how the carer uses the intervention and the ways in which this may reflect the changing needs of the cared-for person and the carer. At regular intervals, the user is prompted to revisit their profile to provide further information that can be used to drive the provision of relevant, personalized notifications. In this way, the intervention aims to prompt and motivate users to access information that is likely to be relevant to their needs but which they might not have considered as being relevant. The importance of addressing “not knowing what you don’t know about” was something that had been emphasized by many carers throughout the development process.

## Discussion

### Principal Findings

Family and other unpaid carers are enormously important to society and yet often feel unsupported and lacking in key knowledge, information, and skills that might make the caring role more sustainable. Current services in the United Kingdom lack accessibility and availability, and Web-based resources and apps are often difficult to discover and may lack relevance. Here, we have described the coproduction of an intervention aimed at helping informal carers maintain their resilience and cope more effectively through gaining access to personalized information, services, and resources. The intervention was developed systematically based on a theory-based behavior change model, existing research, and involvement of multiple stakeholders and carers. Its design reflects the importance of a multicomponent intervention that carers can use flexibly over time according to the changing needs and requirements. Through completing the profile, the intervention can filter relevant information in terms of its applicability to the conditions and context of the cared-for person, and through notifications, the intervention can suggest important or useful actions to be undertaken. In addition, user engagement is encouraged through allowing carers to save resources to a favorites section, adding entries to the journal and making use of a mood monitor. Hitherto, few digital interventions targeting the carer community have used a theoretical basis for intervention development or provided information on how the intervention was developed [16,63-65].

The “person-based” approach that we applied [35] focused on understanding and accommodating the perspectives of carers through synthesizing evidence from the research and policy literature with focus groups and input from carers at every stage of the developmental process. This ensured that the IT designers drew on an in-depth understanding of the biopsychosocial context of carers, and through working to an agile lean methodology could iteratively modify its design to make it more engaging, relevant, applicable, and feasible. In addition, the person-based approach enabled us to identify and highlight the distinctive ways that the intervention has been designed to address key context-specific behavioral issues.

### Strengths and Limitations

A key strength of this study is its participatory approach which included iterative involvement from individual carers, representatives of carer groups, local authority managers, health service commissioners and clinicians, voluntary sector and third-sector organizations, mental health services, clinical and behavioral psychologists, and IT design and software engineers.

Coproduction involving an ongoing collaboration with carers enabled the emergence of in-depth understanding of how carers might use the Care Companion, which in turn influenced each stage of the intervention development. It was informed by theories relating to resilience and coping and the BCW. Together, this allowed a systematic framework in which specific behavioral components to promote resilience and coping could be defined and prioritized. The BCW proved valuable in understanding the capabilities, opportunities, and motivation to maintain coping and resilience as a carer, and helped in the selection of intervention functions and specifying features. It facilitated an extensive range of intervention options to be considered and the eventual definition of an in-depth specification of requirements that were grounded in evidence and the experience of carers.

This required considerable commitment from carers, reinforced by clear demonstration that their views and insights were being directly reflected in the intervention development. The value of this input was recognized with participants being paid an honorarium in line with nationally recommended guidance [66]. In addition, the wide representation of stakeholder organizations that were involved in the development has been a strength that ensured buy-in and support for the future use and dissemination of the intervention.

The use of the BCW framework facilitated the inclusion of contextual and environmental influences into the design of the intervention [35,67]. Limitations were that the BCW was unable to provide guidance on the operationalization of potential intervention components into program features. Hence, it provided a useful starting point and a structure for considering functionality, but much of the design was reliant on the expertise and creativity of the carer and stakeholder groups, including the prudent advice on feasibility and cost from the IT designers, on how to best translate the proposed components into prototype functionality. The challenges faced by informal carers are vast and complex. Specifying a single behavior in terms of who, what, where, and when, as advised by the BCW model, was not appropriate, and rather than the sequential steps in the development, there was a need for a more iterative and overlapping process. However, it enabled us to consider potential intervention features in terms of their relevance, acceptability, and feasibility. There are few evidenced examples of applying the BCW approach to complex behaviors through digital media, and its effectiveness in this context requires further study [68].

### Future Work

The next stage in the intervention development is a feasibility study to test the usability and acceptability of the Care Companion prototype with a wider group of users. This includes the feasibility of peer to peer dissemination of the Care Companion to identify the extent to which peers might be included in the design of a future implementation and adoption strategy. We will be exploring carers’ experience of using the Care Companion, barriers to uptake, and concerns (such as information security) that may limit usage. This work is underway and will be reported in a subsequent publication.

We are also working with commissioners of local services for older people and their carers to define how the wealth of data that could be collected longitudinally by the Care Companion can be used to inform commissioning decisions. Commissioners will have the opportunity to suggest data gathering questions that are locality-specific (ie, only seen by users that are living within these areas) and relevant to commissioning of services, and to define requirements for reporting on the take-up and use of the Care Companion by locality.

### Conclusions

This paper has described how a theory-based approach to intervention development provided a systematic and comprehensive framework for designing a program that addresses a highly complex set of behaviors relevant to caring. The strength of the coproduction described here is its inclusiveness, which ensured that a range of perspectives were iteratively engaged in informing and refining its content and features. As such, it includes many features which might otherwise have not been prioritized for inclusion. The effectiveness of the intervention on carers’ resilience and coping, and the subsequent effect on the cared-for person’s health and well-being, will be studied in future research.

## Appendix

Multimedia Appendix 1 Keywords used in literature search.

## Abbreviations

BCW: Behavior Change Wheel
CCG: Clinical Commissioning Group
IT: information technology
NHS: National Health Service
