# Supplementary material for: Coproduction of a Theory-Based Digital Resource for Unpaid Carers (The Care Companion): Mixed-Methods Study
Source: JMIR Aging. 2018 Feb 28;1(1):e1. doi: 10.2196/aging.9025 (PMC6716079; doi:10.2196/aging.9025)
Supplement: Multimedia Appendix 1 [file aging_v1i1e1_app1.pdf]

Appendix 1 – Key Words used in literature search

Who	Why	How	What
Elderly	Health condition	Technological innovation	Health care
Frail elderly	Long-term health condition	Technological intervention	Integrated care
Aged	Age-related disease	Smart technology	Social care
Ageing	Chronic Illness	Information & Communication Technology	Social support
Aging	Chronic disease	Technology Enhanced Care Service	Social network
Cared-for	Physical disability	Telehealth	Social media
Older person	Learning difficulty	Telecare	Informal support
Older person requiring care	Mental health problem	Tele coaching	Informal support networks
Older adults	Life expectancy	Digital care	Forum
Older people	Hospital admissions	Digital health service	Discussion board
Older population	Needs	Internet-based	Online discussion
Oldest old	Unmet needs	MHealth	Support group
Carer	Caregiver strain	Handheld computer	Voluntary groups
Informal carer	Burden of care	Mini computer	Social agencies
Hidden carer	Quality of life	Tablet	Self-assessment
Spousal carer	Stress	Personal Data Assistant (PDA)	Self-help
Unpaid carer	Self-efficacy	Smartphone	Health-based information
Family carer	Self-esteem	Mobile phone	Health promotion
Family caregiver	Mental health	Android phone	Health literacy
Family member	Social isolation	Mobile device	Legal information
Relative	Wellbeing	Mobile application	Financial Information
Close relative	Loneliness	Mobile phone application	Signpost
	General health	Mobile health application	Online education programme
	Health impact	Smartphone application	Training
	Hostility	Self-care app	Digital Inclusion
		Health app	Digital capability
		Medical app	Social participation
		Smartphone-based intervention	Social engagement
		Mobile health intervention	Empowerment
			Ownership

			Independence
